# Cell-Penetrating Peptides and Transportan

**DOI:** 10.3390/pharmaceutics13070987

**Published:** 2021-06-29

**Authors:** Ülo Langel

**Affiliations:** 1 Department of Biochemistry and Biophysics, Stockholm University, S.Arrheniusv. 16B, Room C466, SE-106 91 Stockholm, Sweden; Ulo.Langel@dbb.su; Tel.: +46-8-161-793 or +46-707-905-284; Fax: +46-8-161-371; 2 Laboratory of Molecular Biotechnology, Institute of Technology, University of Tartu, Nooruse 1, 50411 Tartu, Estonia

**Keywords:** transportan, cell-penetrating peptides, transfection, PepFect, NickFect

## Abstract

In the most recent 25–30 years, multiple novel mechanisms and applications of cell-penetrating peptides (CPP) have been demonstrated, leading to novel drug delivery systems. In this review, I present a brief introduction to the CPP area with selected recent achievements. This is followed by a nostalgic journey into the research in my own laboratories, which lead to multiple CPPs, starting from transportan and paving a way to CPP-based therapeutic developments in the delivery of bio-functional materials, such as peptides, proteins, vaccines, oligonucleotides and small molecules, etc.

## 1. Introduction to Cell-Penetrating Peptides

The recent definition of CPPs [1,2] given below tries to summarize the diffuse diversity of a huge class of peptides with multiple bioactive properties and drug delivery abilities:

Cell-penetrating peptides (CPPs) are relatively short peptides, 4–40 aa, with the ability to gain access to the cell interior by means of different mechanisms, mainly including endocytosis, and/or with the capacity to promote the intracellular effects by these peptides themselves, or by the delivered covalently or non-covalently conjugated bioactive cargoes.

The discovery story of CPPs (also known as protein/peptide transduction domains, PTD, Trojan peptides or shuttling peptides) has been covered in detail [2], starting from the discovery of an HIV tat trans-activator protein [3,4], a membrane shuttling protein with only the portions (residues 1–72 and 37–72) necessary for cellular uptake [5]. The group of Alain Prochiantz [6] introduced the 60 aa homeodomain of Antennapedia (a *Drosophila* homeoprotein), and its short fragment, a 16 aa peptide pAntp(43–58), later named penetratin [7], which I define as a starting point for CPP research. Shortly after this, the group of Bernard Lebleu introduced the short 12 aa Tat peptide [8]. It seems that these first CPPs—penetratin and Tat peptides—are the most popular CPPs today for the trans-barrier delivery of multiple bioactive cargos. These findings introduced a breakthrough situation in cellular biochemistry back in the 1990s, breaking the traditional dogma that the cell plasma membrane was impermeable to proteins and peptides.

To date, June 2021, the website CPPsite 2.0 (http://crdd.osdd.net/raghava/cppsite/) database contains around 1700 unique, experimentally validated CPPs, together with their secondary and tertiary structures. However, in silico CPP predictions show thousands (if not millions) of such peptides awaiting confirmation and application. Most of these CPP sequences can be found in [2], and are not presented here.

Undeniably, the complexities of their mechanisms of action have rendered CPPs problematic to define, if indeed this is possible at all [9]. I have recently even suggested [2] a new way to classify CPPs, based on the multiple sides of CPP activities:

1. Protein-derived vs. designed
2. Classified by physico-chemical properties vs. classified by structural properties
3. Predicted vs. random
4. Linear vs. cyclic
5. Protein mimics vs. cargo delivery vectors
6. Nonspecific vs. targeted
7. “Direct” translocators vs. endocytosis enhancers
8. “Non-toxic” vs. antimicrobial

One can easily see from this CPP classification that the CPP subclasses often overlap, and that many CPPs can belong to several subclasses. Additionally, one can easily create one’s own classification based on different CPP properties; more details are given in [2]. I hope that the work of CPP classification is still in progress today.

For the trans-barrier delivery of different cargos, diverse strategies are used in which covalent conjugation or non-covalent complex formation can be selected [10]. Many examples of CPP/cargo conjugations are available using multiple conjugation chemistries or complexation approaches [2].

CPPs have been extensively employed to transport cargo molecules in vitro and in vivo; however, the delivery uptake mechanism of the particles formed by CPPs and their cargo is poorly understood, depending on, e.g., the membrane structure, the peptide structures, the nature of the cargo, or the concentration of a particle, etc. The knowledge of these mechanisms, however, is the prerequisite for the development of drug delivery systems based on CPP technologies.

Two main types of CPP uptake mechanisms have been suggested: energy-independent (“direct penetration”) and endocytotic pathways. The energy-independent uptake pathway involves CPP/membrane interactions, and can be due to, e.g., pore formation or membrane disturbance, etc. Energy-dependent pathways are usually related to endocytic mechanisms, e.g., macropinocytosis has been shown to be able to incorporate CPPs and their complexes with cargos. Usually, endocytotic uptake is initiated by the interactions of CPPs with different cell-surface receptors, e.g., anionic receptors such as neuropilin-1 and heparan sulfate proteoglycans [9]. The current understanding is that, usually, such a cellular uptake event is the consequence of the parallel action of the above pathways, depending on the conditions.

It is a paradigm in CPP research that the peptides are taken up by virtually all cells, but in vivo CPPs only target a very limited number of cells and many tissues are hardly reached at all. Today’s research aims to target specifically certain cells or diseased tissues for highly efficient CPP-based targeted therapeutics. One research area fueling this research is the need for therapeutics and diagnostics in oncology [11].

All of these aspects (and more) of research of the field of cell-penetrating peptides are often studied for individual CPPs. Additionally, multiple reports are available concerning the comparison of the properties and efficacy of several CPPs in parallel, creating additional information for the development of novel drug delivery systems.

Below, I briefly summarize our work on the introduction and development of one CPP family—transportans—and their further development into a carrier of bioactive molecules as a possibility for future drug development.

## 2. Transportan

### 2.1. Discovery of Transportans, Development of PepFects and NickFects

Galparan, a chimeric peptide, was introduced in 1996 by fusing two naturally occurring aa sequences: amphipathic peptide mastoparan from wasp venom and the fragment of human neuropeptide galanin [12,13]. This fusion peptide was a logical step in our study of an exciting class of our chimeric galanin receptor ligands, developed in Prof. Tamas Bartfai’s group at the beginning of 1990s [14,15]. We were studying the possible rules behind the interactions between galanin receptors and the components of these high-affinity chimeric ligands with peculiar synergistic effects, which were apparently independent of the fused constituents. The rationale behind the fusion of the membrane-active mastoparan (at C-terminus) with galanin(1–13) was the possibility to add a plasma membrane interaction to the interactions with the galanin receptors; see the sequences below.

**Galanin(1–13):** GWTLNSAGYLLG-P [12]

**Mastoparan:** INLKALAALAKKIL [12]

**Galparan:** GWTLNSAGYLLG-P-INLKALAALAKKIL [12]

Galanin receptors in CNS recognized galparan with high affinity, K_D_ = 6.4 nM; however, its bio-effects differed from those of galanin and mastoparan [16]. Galparan induced a 26-fold increase in insulin secretion from rat pancreatic islets at a distal site in the stimulus secretion coupling of the B cell [17], induced in vivo acetylcholine release when injected intracerebroventricularly into the frontal cortex of the rat by not acting at the galanin receptors or at the sites of mastoparan action [18], and modulated the activity of GTPases and Na^+^,K^+^-ATPase, whereas galanin does not affect these enzymes [12,19].

In the hope of understanding galparan’s interactions with plasma membrane-located galanin receptors, we further modified it by the replacement of P^13^ with K^13^. The novel peptide, called transportan, showed, to our surprise, the translocation to the cell cytosol when labelled at the K^13^ side chain with a fluorophore, which was an early experiment carried out by Margus Pooga [20]. Systematic structure-activity studies enabled the shortening of the transportan to transportan 10, which demonstrated excellent cellular translocation with decreased toxicity [21]. Hence, the discovery of transportan is a good example of serendipity in research; however, it was made possible only due to the specific exciting questions asked in the study.

**Transportan, TP:** GWTLNSAGYLLG-K*-INLKALAALAKKIL [20]

**Transportan 10, TP10:** AGYLLG-K*-INLKALAALAKKIL [21]

Later, the K^13^ side chain modification was used for the covalent modification of several cargo molecules, e.g., biotin, PNA and proteins bound to biotin, etc., see below.

Interestingly and independently, several additional chimeric peptides were reported to obtain CPP properties, e.g., MPG and Pep-1, combining the nuclear localisation signal (NLS) of SV40 T-antigen and a hydrophobic peptide with high affinity to cellular membranes [22,23], CADY [24] and Pip (PNA-internalising peptides) [25], all successful “examples for such design where cationic, hydrophobic and amphipathic sequences have been combined” [26].

The further development of transportan analogs yielded a novel CPP series, introduced by my Stockholm and Tartu groups, respectively, PepFects and NickFects, exemplified by PF6, PF14, NF51 and NF55. PepFect strategies were protected by a patent application (publication number: 20140140929, together with CePeP and General Electrics Healthcare). NickFects were protected by a patent application (PCT/EP11155275.8, WO 2012/113846 Al) and further developed by CePeP, Sweden/Estonia, PepFex, Sweden and Tartu University, leading to an additional patent application (PCT/EP2020/050524) to protect the third generation NickFects, developed by Tartu University.

In PepFect6, the side chain of Lys* was modified with branched four chloroquine analogs, hopefully enabling improved endosomal escape for PepFect/cargo. PepFect14, designed by Mattias Hällbrink, consists of the same N-terminus as PepFect6, but with an ornithine (O) containing an (educated) fantasy sequence in the C-terminus, leading also to endosomal escape similar to PF6.

**PepFect 6, PF6:** stearoyl-AGYLLG-K*-INLKALAALAKKIL [27]

**PepFect 14, PF14:** stearoyl-AGYLLG-K*-LLOOLAAAALOOLL [28]

**NickFect 51, NF51:** O(Nδ-stearoyl-AGYLLG)-INLKALAALAKKIL [29]

**NickFect 55, NF55:** O(Nδ-stearoyl-AGYLLG)-INLKALAALAKAIL [30]

While the PepFect vectors contain linear aa sequences, the NickFect peptides contain the branched structure at the side chain of ornithine, although the original sequences from galanin and mastoparan are still present. Such a branched structure in the transfection vectors yielded several CPPs with improved properties, i.e., lower toxicity and the improved tranfection of oligonucleotides, depending on different delivery systems. The mechanisms of such improvements are not clear and the current research addresses these questions intensively. One important difference between “traditional” CPPs and PepFect or NickFect vectors is that the latter form nanoparticles with oligonucleotide cargos; see below.

### 2.2. Antisense ON, siRNA and Plasmid Delivery by Covalent Coupling

Here, I summarize the transportan delivery of cargo (and not the delivery by ON/CPP nanoparticles); see also the earlier reviews [31,32]. The first intracellular CPP delivery of antisense ONs (ASO) was achieved in 1995 by the covalent disulfide conjugate of penetratin (then called pAntp) with phosphorothioate ONs blocking APP expression [33]. This was followed by intensive applications of CPPs in antisense ON transfection by covalent conjugation in the 1990s and, more recently, by non-covalent conjugation when the respective technologies became available; see below. Today, the transfection of “traditional” antisense ONs has been reported for multiple CPPs, e.g., penetratin, Tat, Pip, (KFFK)_3_R, (RXR)_4_, Pep-3, MPG, R15, TP10, PepFects, NickFects, and Chol-R_9,_ etc., in a covalent or non-covalent manner, in vivo and in vitro, as summarized in [2].

Our group’s initial interest in vectors for the intracellular delivery of cargos was fueled by the introduction of cell-membrane-impermeable peptide nucleic acids (PNA) by a Danish group [34]. PNA oligomers with extraordinary properties, such as a high affinity to complementary DNA or RNA, and high resistance to the protease or nuclease degradation, etc., were potential intracellular regulators of DNA/RNA-initiated processes. Later, this was shown to be true after solving the main hurdles with their intracellular delivery.

After the finding of the cargo delivery properties of transportan, our group, in 1998, (patented 1997 [35], together with PerSeptive Biosystems Inc., USA, by the initiative of Michael Egholm) conjugated it (and penetratin) with a PNA oligomer via the disulfide bridge, yielding a covalent conjugate, CPP-S-S-PNA. The conjugate was designed to be reduced by intracellular glutathione, and to liberate the PNA oligomer to knock down galanin receptor 1 expression by the antisense (translational arrest) mechanism. Both constructs were successfully internalized into Bowes cells and knocked down targeted galanin receptor type 1 [36] in vitro and (intrathecally) in vivo, decreasing the galanin binding in the dorsal horn, and the inability of galanin to inhibit the C fiber stimulation-induced facilitation of the rat flexor reflex [36,37]. It seems that disulfide-based conjugates are convenient in, at least, the delivery of CPP-PNA conjugates. Several reports are available on the applications of covalent transportan-ASO and other drug conjugates.

Transportan-S-S-PNA (antisense to PTP sigma) increased the glucose-induced insulin secretion from GK rat islets, with decreased amounts of phosphatase [38]. The liposomes modified with a transportan10 analog, TH, showed enhanced cellular uptake and the delivery of paclitaxel with the inhibition of tumor cell growth in vivo [39]. The uptake into human fibroblast cytosolic compartments was seen for Tat, penetratin, R9F2 and transportan disulfide conjugates of 12mer OMe/LNA oligonucleotide conjugates targeted to the TAR RNA, in HeLa and human fibroblast cells [40]. Disulphide-conjugated penetratin, Tat, transportan, transportan-21 and transportan-22 to a 16-mer PNA, targeting the TAR region of the HIV-1 genome, showed cellular uptake and anti-HIV virucidal activity by the inhibition of HIV-1 replication in vitro [41]. Transportan10-S-S-PNA-Bpa (*p*-benzoylphenylalanine), targeting the regions of the 3′ and 5′ UTRs of ankylosis mRNA, showed intracellular crosslinking to RNA-binding proteins (RBPs) that complex with a target RNA in vivo. Several proteins were isolated and identified “in complex with or near the targeted regions of the ankylosis mRNA through UV-induced crosslinking of the annealed PNA-RNA-RBP complex” [42]. Transportan10-S-S-PNA-based antisense conjugate was used to study “the role of subtypes of the L-type voltage-gated calcium channels (LTCs), Ca(V)1.2 and Ca(V)1.3 in long-term pain sensitization in a rat model of neuropathy”, showing the reverse of the neuropathy-associated mechanical hypersensitivity and the hyperexcitability, as confirmed with siRNA knock-down experiments [43]. Transportan-PNA_TAR_ internalized into the cells with the functional inhibition of HIV-1 production in chronically HIV-1-infected H9 cells [44].

An additional antisense ON application field became routinely available after R.Kole and co-workers introduced a novel antisense ON-based platform to redirect splicing by blocking aberrant splice sites in HeLa cells stably expressing luciferase containing such a site [45]. This system of splice correction by ASO is a promising therapeutic tool for a variety of diseases, e.g., Duchenne muscular dystrophy, beta-thalassemia, cystic fibrosis and certain cancers.

We applied the luciferase aberrant splice site setup in HeLa cells with known splice-correcting PNAs (PNA705), tethered to a variety of CPPs [46], Tat, penetratin and transportan, via a disulfide bridge. Transportan was the most potent vector, and it significantly restored splicing in a concentration-dependent manner, following the endocytotic cellular uptake. This suggested that CPPs can be used for the delivery of splice-correcting ASO as a potential therapeutic approach for the regulation of splicing in a variety of diseases.

Since then, the HeLa based delivery of a splice-correcting CPP-ASO assay has been in routine use in the evaluation of novel CPPs for cargo delivery and studies of internalization mechanisms, e.g., to assess different endocytic pathways and the dependence on extracellular heparan sulfates for internalization for the comparison of penetratin, Tat, transportan, TP10, MAP and pVEC [47], showing the exact endocytic internalization routes.

Disulfide-linked CPP conjugates with oligonucleotide analogues, siRNA and PNA, in the HeLa cell assay with integrated plasmid reporters showed that transportan-PNA and R6-penetratin-S-S-PNA caused the Tat-dependent trans-activation inhibition, suggesting them as potential anti-HIV agents [48].

Seven different CPPs were included in the study: transportan, R7–9, Tat, penetratin, KFF, SynB3 and NLS, conjugated by different conjugation chemistries. PNA-S-S-transportan-amide (ortho)-PNA, targeting luciferase expression correction, was the most potent conjugate, resulting in the maximum luciferase signal in the serum-free media, but it was the least sensitive to the presence of serum [49].

The development of antisense ONs (mainly PNA, LNA, mixomers, PS and PMO) in splicing redirection using CPP transfection is very active [50,51,52]. For example, Sarepta (Cambridge, MA, USA) works intensively with RNA-targeted therapeutic candidates for different types of RNA, using phosphorodiamidate morpholino oligomers (PMOs) as antisense ONs, attached to CPPs (PPMO) such as (R-Ahx-R)_4_, (6-aminohexanoic acid-spaced oligo-arginine). The development of next-generation PMO-based chemistries for advanced RNA-targeted therapeutics with enhanced tissue targeting, intracellular delivery, target selectivity and drug potency is in progress [53].

PNA was designed to interact with an overlap of the NFkappaB decoy oligonucleotide consisting “of a double-stranded consensus sequence corresponding to the kappaB site localized in the IL-6 gene promoter”. It was shown that the construct “blocked the effect of interleukin-1beta-induced NFkappaB activation and IL-6 gene expression” [54].

Few examples are available of applications of CPP-siRNA covalent conjugates for the knockdown of gene expression. Our group was never successful with this knock-down strategy. We often achieved the cellular internalization of the CPP-S-S-siRNA conjugates; however, the functional knockdown was never demonstrated in different cells. Interestingly, the covalent CPP (penetratin and transportan) coupling of siRNA via disulfide bond was carried out, yielding an improvement of the cellular uptake as well as the expression reduction of reporter GFP transgenes [55]. Conjugates of transportan10-S-S-siRNA showed intracellular localization and silencing by siRNA-targeted firefly luciferase GL3 in FRSK cells [56]. Transportan or transportan-r9, T9(dR), complexed with siRNA against a nucleoprotein (NP) gene segment of the influenza virus (siNP) showed the in vivo and in vitro delivery of siRNA in 293T, MDCK, RAW and A549 cells and mice. After the combined tail vein injection of siNP and T9(dR) or transportan, all of the mice “infected with PR8 influenza virus survived and showed weight recovery at 2 weeks post-infection” [57].

The improvement of intracellular plasmid delivery by CPPs has been a desirable objective for several years. Few reports are available on plasmid delivery by transportans; however, these have a low success rate, indicating the need for additional CPP vector development for efficient plasmid transfections. Transportan10 “crosslinked to a plasmid via a PNA oligomer, TP10 conjugation with polyethyleneimine (PEI), and addition of unconjugated TP10 to standard PEI transfection assay” increases the transfection efficiency several fold compared to PEI alone in Neuro-2a cells [58]. Fl-Transportan showed the maximum fluorescence among all of the tested CPPs in permebilized wheat immature embryos [59]. While Tat(2) mediated the GUS enzyme and plasmid DNA (carrying Act-1GUS) delivery to embryos, transfection success with transportan was not reported [59]. Stearoyl-TP10 was shown to form stable nanoparticles with plasmids that efficiently enter different cell-types, including primary cells, resulting in a gene expression which was almost comparable with the levels of Lipofectamine 2000 (LF2000) in vitro, and which mediates efficient gene delivery in vivo when administrated intramuscularly (i.m.) or intradermally (i.d.) in mice [60]. This work enabled us to further aim at the development of non-covalently conjugated plasmid transfection strategies.

### 2.3. Delivery of Peptides and Proteins

The conjugation of several CPPs to proteins and peptides has improved their rapid translocation into cells and through biobarriers in vivo [31,61,62,63]. The obvious CPP champions in protein trans-barrier delivery are Tat and penetratin; however, several reports are available on transportan conjugates with proteins and peptides, and their delivery. Besides the obvious therapeutic task with improved protein delivery, these cell-permeable proteins serve as valuable tools for the clarification of the uptake and targeting mechanisms for CPP-cargos; see below.

#### 2.3.1. Peptides

A few examples can be found on the transportan delivery of short peptides; see below. Although efficient, the addition of transportan to the peptides yields a much longer peptide, making its production more difficult. Hence, the preferred choice for the cellular delivery of short peptides would be to use the CPP prediction-based modifications of the selected peptides.

The cellular uptake and cargo delivery kinetics were studied for penetratin, transportan, Tat and MAP, labelled with the fluorescence quencher 3-nitrotyrosine, coupled via disulfide to a pentapeptide cargo (labelled with the 2-amino benzoic acid fluorophore) [64]. The DOCK2 inhibitory peptides for protein–protein interaction conjugated to 13 different luciferin-conjugated CPPs, among them transportan, to test them as an “intracellular target for transplant rejection and inflammatory diseases” [65]. Several synthetic peptides comprised from effector caspase activational cleavage sequences fused with Tat, penetratin, transportan, and Pep1 showed the internalization and improved survival of syngeneic immortalized Schwann cells during transplantation in vivo [66]. Transportan-Aβ42 peptide showed tissue penetrating capability, and was introduced into the adult zebrafish brain [67]. Transportan (and other CPP’s) conjugated with PKI and NBD peptides showed cellular uptake and its time course [68].

#### 2.3.2. Proteins

Several initial attempts for intracellular protein delivery by CPPs have been carried out using the strong biotin–avidin interaction for the non-covalent conjugation of biotinyl-CPPs and (strept)avidin or biotin antibodies [69]. In the case of transportan, the following combinations were used: biotinyl–transportan with anti-biotin monoclonal antibodies [70]; an Nα-biotinyl-TP10 complex with fluorescently labeled streptavidin in a photo-induced endosomal escape study [71]; biotinyl–transportan, -oligoarginine and -Tat, complexed to avidin–TexasRed, showing three different populations of complexes-containing vesicles [72]; biotinyl–penetratin, Tat, transportan and pVEC complexed with avidin, showed endocytotic and clathrin-dependent and -independent mechanisms of cellular transduction [73]; colloidal gold-labeled neutravidin complexes with biotinyl-transportan and nanogold-labeled peptides showed endocytotic routes in parallel translocation [69,74,75]; biotinyl-Tat/streptavidin conjugated to a biotinylated, pH-responsive polymer poly(propylacrylic acid) showed improved endosomal escape [74]; biotin–transportan and -TP10 showed an endocytosis-independent mechanism in colorectal cancer (CRC) cells [75]; biotinyl–penetratin, -Tat and -transportan 10 with avidin and streptavidin showed that the cellular delivery properties are dependent on the cargo used [76]; and biotinyl–transportan and -Tat complexed with avidin-β-galactosidase (ABG) showed enhanced tissue distribution in samples of freshly harvested human carcinoma or hyperplasia-containing specimens of the uterus and the cervix [77]. Colorectal cancer (CRC) cell lines HT29 and HCT116, incubated with siRNA for SASH1 mRNA in the presence of transportan and TP10, showed cellular uptake and a decreased SASH1 mRNA level [75].

A few reports with no use of biotin/avidin interaction are available on the cellular delivery of proteins by transportans. Tat, penetratin and transportan complexed with rhodamine-BSA, which showed delivery to the interior of epithelial cells, being “passive carriers that do not initiate epithelial cell-associated ‘danger signals’ during the process of cytoplasmic delivery of a model protein cargo” [78]. Transportan-GFP and antibodies could internalize covalently coupled molecules up to 150 kDa in Bowes cells [70]. Penetratin showed the cellular uptake of complexed BSA, while R8 and TP10 failed to deliver BSA [79]. GFP fused to penetratin, R8, Tat, transportan, Xentry and their cyclic derivatives showed cellular uptake with localization in endosomes in human cell lines HeLa, HEK, 10T1/2 and HepG2 [80].

### 2.4. Complexation, PepFects and NickFects

As described above, transportan and its modified versions have been demonstrated to aid efficiently the cellular internalization of a variety of covalently conjugated bioactive cargos, both in vitro and sometimes even in vivo. Often, these examples are available as comparisons with additional CPPs to transportan. However, our routine testing of multiple CPPs for their cargo delivery capacity was hindered by the need for the covalent conjugation of CPP with cargo due to the additional steps of chemistry and purification/characterization. Our attempts to attach unmodified transportan non-covalently to several cargos were mainly unsuccessful.

Hence, we further aimed to find non-covalent transportan/cargo conjugation methods which could provide strategies of the simple complexation of CPPs with different cargos yielding efficient cargo delivery strategies. The non-covalent complex formation of CPPs with ONs was an obvious choice on first sight due to the availability of cationic CPPs and anionic ONs, enabling the formation of efficient and stable aggregates or even nanoparticles, possibly yielding efficient transfections. The first CPPs for non-covalent ON complexation were stearoyl-R9, GALA, KALA, MPG, Pep-1, CADY [81,82,83,84,85,86,87,88], Chol-R9 [89], stearoyl-(RXR)_4_ [90] and others.

Our first choice in the case of transportan was to test stearoyl–transportan [27] for ON transfection, based on the idea of Prof. Shiroh Futaki’s group on stearoyl-R9 [81]. We tested multiple fatty-acid-modified transportan analogs, and now the series of PepFects (PF) and NickFects (NF) are available; see above. The PepFects and NickFects are excellent ON delivery vectors, as exemplified in multiple reports below. They form relatively stable nanocomplexes with ONs, enabling ON transfection in vitro and in vivo by the non-covalent simple formulation technology of antisense, siRNA and plasmid delivery [27,28,29,90,91,92,93,94].

Due to the formation of these nanocomplexes, it has even been questioned whether PepFects and NickFects are the “true” cell-penetrating peptides, rather than the new peptide-based detergents. My answer is a clear “yes”, as they contain stearoyl-modified transportan, and the yielding CPP is a cargo delivery vector, following the rules for the CPP definition, even if they may obtain detergent-like properties. The mechanisms of such transfections are discussed below.

#### 2.4.1. PepFects

Several reports are available on the design of the PepFect and NickFect peptides. Novel PFs and NFs were introduced using a QSAR prediction model, showing peptide-plasmid complexes and the transfection of cells with pDNA [95]. PepFect analogues with introduced His residues were introduced in order to make the peptides pH-responsive for the PepFect/SCO nanocomplexes, showing PepFect132 with high bioactivity [96]. PepFect14, double-functionalized with PEG and an MMP substrate site, complexed with pDNA, showed the efficient induction of gene expression specifically in tumors after i.v. injections [97].

Several examples of myristoyl–transportan transfection are available: NPs incorporating myristoyl–transportan and tumor-homing peptides carrying siRNA, a CpG DNA ligand of TLR9 suppressed tumor growth in several animal models of various cancers after systemic intravenous (i.v.) administration [98]; myristoyl–transportan conjugated to a transferrin receptor-targeting peptide (myr-TP-Tf) encapsulating siRNA targeted it to the brain with a functional gene silencing effect in a human glioma [99]; and a nanocomplex based on a tandem peptide of myristoyl–transportan and Lyp-1 showed the internalization of sgRNA/Cas9 ribonucleoprotein complexes and genome editing in cell lines [100].

Examples of ON cargo delivery with PFs and NFs are available. An antisense nanoprobe, ^99m^Tc-anti-miRNA ONs/PepFect6, was used “for imaging the miRNA-21 expression in A549 lung adenocarcinoma xenografts and in vivo” [101]. The master regulator proteins in critical tumor regulation were confirmed using their lentivirus-mediated shRNA silencing with PF14 transfection, yielding sixteen master regulators which could be reproducibly silenced; of these, 94% showed reduced tumor growth/viability in vitro [102].

PF14/ASO (triplet repeat-targeting AS ON) in a muscle cell model of myotonic dystrophy yielded a dose-dependent correction of disease-typical abnormal splicing, whereas PF14 shielded the AS ON from degradation. It was shown that “intranuclear blocking-type oligonucleotide concentrations in the upper nanomolar range were required to dissolve nuclear muscleblind-like protein 1 foci” [103].

PF14/mRNA nanoparticles showed the expression of reporter protein eGFP “in two-dimensional tissue cultures and in three-dimensional cancer cell spheroids”, as well as in primary ovarian cancer explants [104].

PF14/mRNA (eGFP) complexes in the glomerular endothelial cell line mGEnC, HeLa cells and SKOV-3 ovarian carcinoma cells showed uptake and protein expression with “linear correlation of dose, uptake, and expression, observed over 5 orders of magnitude in vitro and 3 orders of magnitude in vivo” [105].

The PepFects in complex with graphene oxide and plasmids, splice correction oligonucleotides and siRNA showed NPs and a >10–25 fold increase of their cell transfection [106]. Similar effects were achieved with magnetic nanoparticles [107], zeolitic imidazolate frameworks [108] and carbonized-chitosan-encapsulated hierarchical porous zeolitic imidazolate frameworks [109].

Two reports are available on the PepFect transfection of peptides and proteins. Calcium signal activity was tested following the application of a hemichannel blocking peptide, Gap19 (nine aa from connexin 43 cytoplasmic loop), complexed with PF6, showing the reduction of astrocyte response amplitudes and the proportion of ^SE^astrocytes to the EtOH treatment in enriched astrocyte cultures [110]. Nanoparticles of PepFect14 complexed with Heat Shock Protein (HSP70), suggested first by docking [111], showed delivery into Bomirsky Hamster Melanoma cells; this protocol is shown in Falato et al., 2021 (in preparation).

#### 2.4.2. NickFects

PepFect and NickFect supported the delivery of nanocomplexes of Fl-miRNA mimics (NF-miR-146a) into keratinocytes and dendritic cells with the downregulation of miR-146a-influenced genes by endocytosis, as well as suppressing inflammatory responses in a mouse model of irritant contact dermatitis [112].

By modifying the net charge and the helicity of the NickFects, a novel NF55 was introduced, showing in vivo DNA nanoparticle delivery with efficient gene induction in healthy mice, and showing tumor transfection in various mouse tumor models, e.g., an intracranial glioblastoma model [30,113].

The quantitative tracking of NickFect/pDNA complexes through endosomal transport was shown by the separation of endosomal vesicles by differential centrifugation and single-particle tracking using fluorescently labeled cargo and GFP expressing cells. It was shown that NF51 facilitates the rapid internalization of complexes into the cells, prolongs their stay in early endosomes and promotes the release to cytosol [30].

The cellular uptake and NickFect1- and NickFect51-mediated ON delivery were analyzed. It was shown that the “pathway for cellular uptake of peptide complexes is cargo dependent, whereas the endosomal escape efficacy depends on peptide hydrophobicity and chemical structure” [114].

NF55/pDNA nanoparticles showed promising tumor transfection in various mice tumor models, including an intracranial glioblastoma model [30,115].

## 3. Mechanisms

The mechanisms of the uptake of CPPs and their conjugates with bioactive cargos have not yet been clarified due to the availability of multiple diverse CPPs and examples of multiple CPP-cargos for delivery in order to introduce novel therapeutic entities for new pharmacology. This situation has caused constant interest in the characterization of CPP uptake mechanisms with all of the available strategies of chemistry and biology.

The mechanisms of the uptake of transportan and its modifications are not exceptions; one can find attempts to understand its trans-membrane delivery with multiple methods in comparison with several other CPPs [116,117]; see Table 1 and the detailed examples below.

### 3.1. Visualization

Multiple visualization and imaging methods are available to determine the distribution and translocation mechanisms of CPPs in vitro and in vivo [2]. These methods enable us to understand where and how the (drug) molecules are internalized into cells and different organs. In the case of transportan, often in comparison with other CPPs, most of these methods have been utilized; below, a brief selection is presented.

In the CPP labeling for the visualization, it should be considered that the “pharmacophoric” amino acids should not be modified due to their possible functionality, e.g., primary amino groups in the CPPs. Our initial naïve understanding of the transportan uptake mechanisms suggested the application of the side chain of Lys13 for the attachment of biotinyl and fluoresceinyl moieties, as “such modification did not compromise the transportan’s internalization properties”. The introduction of an extra amino acid into the CPP sequence, e.g., Cys, has been used often as a point for cargo attachment.

In the first confocal fluorescence microscopy study, we tested the uptake of N-ε13-biotinyl-transportan in live Bowes cells, then the visualization was carried out on the fixated cells after incubation with streptavidin-FITC or streptavidin-Texas red [20,36]. This method was used for a while, but it is not popular any more after a few reports indicated that, for some CPPs, the fixation could influence the cellular translocation by itself, e.g., in the case of VP22 and Tat [154,155,156]. Hence, the live cell visualization of CPP uptake is today usually applied, although in our hands the information obtained with the fixated cells often coincide with the later live cell studies [73,157].

For example, the ^125^I-transportan uptake studies additionally confirmed that the cellular internalization of transportan is not an artefact caused by cell fixation [20]. The kinetics of internalization showed that “the maximal intracellular concentration is reached in about 20 min at 37 °C”.

Biotinyl-, Fl-, Abz-transportan, -transportan10 and -penetratin were visualized “to cross a Caco-2 human colon cancer cell layer in vitro in Transwell model, showing that transportan peptides pass the epithelial cell layer”, mainly by a transcellular pathway [118].

Fl-transportan antisense conjugates with luciferase splice-correcting ONs were shown by confocal microscopy to be mainly endocytic, in particular the macropinocytotic pathway for cellular uptake [46].

Protein uptake by penetratin, Tat, transportan, and pVEC studies by FACS and spectrofluorimetry showed the endocytotic mechanism in HeLa cells of CPP-avidin complexes [73].

We studied the internalization efficiency of Fl-CPPs, transportan, TP10, penetratin and pVEC, with fluorescence microscopy, spectrofluorometry and FACS in different mammalian and plant cells [119].

Tat, transportan and polyarginine were shown by FACS analysis to have similar kinetic uptake profiles in HeLa, A549 and CHO cell lines [68].

A ^111^In- or ^68^Ga-CPPs (transportan and nine additional CPPs) study by micro-PET imaging showed uptake by six tumor cell lines, biodistribution in PC-3 tumor-bearing nude mice showed accumulation in well-perfused organs, suggesting that “data reveal that CPPs do not show evidence for application in tumor targeting purposes in vivo” if they are not targeted [120].

A fluorescence polarization, quenching and CD spectroscopy study in small phospholipid vesicles showed that the helical penetratin and transportan lie along the vesicle’s surface; penetratin variants appear to penetrate deeper into the membrane [121].

With TEM, the cell entry mechanisms and intracellular trafficking of gold nanoparticle-functionalized transportan- and TP10-protein constructs were studied [122].

Myristoylated transportan, modified with a transferrin receptor-targeting peptide (myr-TP-Tf), encapsulating siRNA showed the targeting of siRNA over BBB; fluorescence images indicated that the siRNA uptake in murine brain endothelioma and a human glioma cell line, and functional gene silencing in “a human glioma cell line as well as in primary murine neurons/astrocytes” [99].

Complexes of TAMRA-transportan 10 or -PTD4 with cisplatin (cPt) in HEK293, HEL299 and HeLa OS143B cell lines were visualized by fluorescence microscopy and inverted phase contrast microscopy, showing TAMRA-TP10 or TAMRA-TP10 + cPt in the interior of the HeLa cells, but not in the non-cancer cells HEK293 and HEL299. Only TP10 improved the anticancer activity of cisplatin if both compounds were used in the form of a complex [123].

Alexa488-labeled R8, penetratin and TP10, shown by flow cytometry, live-cell imaging and image analysis, demonstrated that the glycine–phenylalanine switch was most dramatic in TP10 [79].

The in vivo injection of CPPs into the right jugular externalis vein of anesthetized ICR-CD-1 mice showed the BBB delivery of ^125^I-pVEC, -SynB3, -Tat, -transportan 10 (TP10) and -TP10-2 with a negligible-to-low brain influx by transportan analogs, while Tat, SynB3 and pVEC showed very high unidirectional influx rates; 80% of the influxed peptides reached the brain parenchyma. The CPPs (except pVEC) showed a significant efflux out of the brain [124].

The macrocyclic analogue M13 of transportan-10 showed increased delivery across a cellular spheroid model of the blood–brain barrier; the ex vivo imaging of mouse brains showed the increased penetration of the brain parenchyma following i.v. administration in mice [125].

Penetratin and transportan conjugates with isoniazid (INH, antibacterial agent against tuberculosis, Mtb) showed a similar internalization rate into EBC-1 human squamous cell carcinoma, and a markedly different subcellular localization and activity on intracellular Mtb by the Langmuir balance technique and AFM imaging of the penetrated lipid layers [124].

### 3.2. Structure and Interactions of Transportan and Its Modifications

Knowing the interactions of CPPs with model membranes or lipid bilayers as well as the structures helps to understand their trans-barrier translocation, as studied by different biophysical methods and model systems, e.g., large or giant phospholipid vesicles (LUVs, GUVs) or SDS micelles, etc. [2,158], which have been used to characterize even transportan and its modified versions, often in comparison.

In general, it seems that transportan shows nearly no structure in water and an induced helix in the presence of different micelles. As shown by the CD studies, transportan obtained almost a random secondary coil structure in water, turning in SDS micelles to a 60% induced helix, localized to the C-terminal part of the peptide [127]. Penetratin, pIsl and transportan showed secondary structures of up to a 60% alpha-helix in the presence of various phospholipid vesicles [128]. Transportan showed an induced helical structure in small phospholipid vesicles of varying charge densities. Penetratin interacted only with negatively charged vesicles, and the induced secondary structure depends on the membrane charge and lipid/peptide ratio [121]. The NMR solution structure and the position of the transportan in neutral bicelles showed “the alpha-helix in the C-terminus of the peptide and a weaker tendency to form an alpha-helix in the N-terminal domain, obtaining the parallel to the membrane surface structure” [129]. The structures of transportan in neutral DMPC bicelles and partly negatively charged DMPG-containing bicelles were found to be different from each other [130]. Penetratin, MSI-103, transportan, MAP, SAP, Pep-1 and AMPs were shown to obtain different structures in aqueous solutions, obtaining an amphiphilic α-helix when bound to membranes of vesicles composed of typical eukaryotic lipids [131]. Transportan 10 showed a range of conformations in the DMPC/DMPG vesicle membrane-bound state; the C-terminal α-helix is embedded in the membrane being tilted [132]. Transportan 10 and its five analogs were shown to form an α-helical conformation; the higher membrane disturbance yields a higher cellular uptake in Hela and NIH-3T3 cells [133].

A few reports are available on the characterization of the interactions of transportan with different lipid membranes. CD spectroscopy showed that the secondary structure of transportan and penetratin, isoniazid–penetratin and –transportan showed increased membrane affinity with DPPC and DPPC + mycolic acid mixed monolayers [126]. The binding of transportan 10 and its four variants to phospholipid vesicles showed the peptide-induced efflux, “becoming faster as the Gibbs energies for binding and insertion of the TP10 variants decrease” [134]. Penetratin, transportan and their variants were studied by an algorithmic method named PepLook to analyze their peptide polymorphism, showing common conformational polymorphic characteristics [135]. A cys-transportan interaction study with model DMPC membranes with moderate cholesterol concentrations by EPR showed that Cys-TP caused lipid ordering in the membranes and a large increase in the permeation of DMPC membranes. At a high cholesterol content, the effect of Cys-TP was observed, either on the membrane structure or on the membrane permeability [136]. A molecular dynamics study of the interactions between transportan 10 and a zwitterionic POPC bilayer showed the adoption of an α-helical structure on the membrane surface and binding to the membrane surface with its hydrophobic side facing the hydrophobic lipid core: “the Lys-phosphate salt bridge is a key factor in determining the orientation of the peptide in the interfacial region and the phosphate groups is also believed to be the main bottleneck for the translocation of TP10 across the membrane” [137]. The interaction of GPMVs with six Fl- CPPs (R9, Tat, penetratin, MAP, transportan and TP10) in a model system revealed that amphipathic CPPs preferentially associate with liquid-disordered membrane areas, and all of the tested CPPs accumulated into the lumen of GPMVs [138].

These studies of the structure and interactions of transportan were obtained mainly by using artificial lipid membranes, which only partly mimic the natural membranes of interest, producing the initial and necessary information for the understanding of the processes in cells and tissues.

The introduction of the PepFect (PF) and NickFect (NF) series of very efficient ON delivery vectors (see above) brought us to the need to understand their properties. To start, it was shown that PF6/siRNA [27] and PF14/SCO [28,159] formed complexes (nanoparticles) with ONs, promoting their functional cellular uptake.

The exact structures of such nanoparticles are currently being studied and, hopefully, will be characterized in order to obtain more efficient transfection vectors in the future. It is obvious that the complex nature of the components of the nanoparticles is determined by all of the possible interactions in the complex, such as electrostatic and hydrophobic interactions in the presence of a solvent (water) and its components (salts, etc.). Several examples are available concerning these studies.

The mean diameter of PF6/siRNA nanoparticles was shown to be below 200 nm [27]. DLS studies of PF14/SCO nanocomplexes as the solid formulations showed “that the particles size and particle-size distribution is highly affected by the type of excipient”, and that, to our surprise, the nanocomplexes—with or without serum—possessed a net negative charge [28]. In order to improve the PepFect vectors, the peptides/Luc-plasmid complexes were studied by CD spectroscopy, DLS and a quantitative structure–activity relationship model using descriptors including hydrogen bonding, the peptide charge and the positions of nitrogen atoms. The cellular uptake data was correlated to QSAR predictions, improving the understanding of the structural requirements for cell penetration [95]. In order to make the peptides pH-responsive in an SCO assay, His-modified PepFects were introduced and characterized by DLS and CD spectroscopy. The membrane interactions in large unilammelar vesicles were studied using a calcein leakage assay. The complexes formed were small at pH 7 and grew under acidic conditions. The most promising PepFect, PepFect 132, has a significantly higher bioactivity and membrane activity [96].

### 3.3. Kinetics

Studies of the CPP translocation kinetics obviously add to the understanding of the internalization mechanisms. Unfortunately, only a few kinetics studies are available on CPP uptake pathways and, hence, the careful characterization of the complicated multi-step internalization is not available today. One summary of such hypothetical processes is presented in [2], in which the “free”, “bound” or metabolized, etc., states of CPPs exist, likely in equilibrum. To simplify, first-order kinetics have been adopted to study CPP translocation [160]. Furthermore, the smaller cargoes seem not to influence the rate of internalization, but larger cargoes significantly slowed this rate [76,161].

Few reports are available concerning the internalization kinetics of transportan, often concerning artificial lipid membranes of different types. Hopefully, these studies will add to the general knowledge about CPP uptake mechanisms. Additionally, the difficulty in these studies is that very little is known about the kinetics of the uptake of the transportan/cargo conjugates.

#### 3.3.1. Cells

The uptake kinetics of ^125^I-transportan (with iodinated side-chains of Tyr by the chloramine T method) in Bowes melanoma cells “where, at 37 °C, the maximal intracellular concentration was reached in about 20 min (t_1/2_ of 3–4 min)” [20].

A 2-amino benzoic acid fluorophore-modified pentapeptide conjugated by disulfide to 3-nitrotyrosine-penetratin, -transportan, -Tat and -MAP showed the cellular uptake of the cargo as an increase in fluorescence intensity when the disulfide bond of the CPP-S-S-cargo construct was reduced in the intracellular milieu [64].

Three CPPs—M918, TP10 and pVec—used a quenched fluorescence assay with the fluorophore Abz and His(DNP) as a quencher for uptake kinetics studies [162].

The uptake kinetics of PNA_TAR_-^125^I -transportan conjugate showed “a sigmoidal curve with a cooperativity index of 6, indicating very rapid cellular uptake” by the receptor-independent endocytotic pathway in Jurkat cells. The [*S*]_0.5_ value and cooperativity index determined from the sigmoidal plot were 1.5 and 6 μM, respectively, with a Hill coefficient of 0.53 “suggesting that the observed cooperativity is not due to multiple conjugate binding sites on the membrane” [143].

Penetratin, Tat, transportan and polyarginine demonstrate similar kinetic uptake profiles according to FACS analysis, “being maximal at 1–3 h and independent of cell type (HeLa, A549 and CHO cell lines)”. The time course of the uptake and their cellular distribution did not correlate with transferrin, a marker of clathrin-mediated endocytosis, but the peptides co-localised with a marker of the lipid raft domains, cholera toxin [68].

The uptake kinetics of ^125^I-labeled Fl-PNA_Tar_-conjugated penetratin, Tat, transportan-27, transportan-21 and transportan-22 showed a sigmoidal curve, “suggesting a cooperative interaction between the conjugate and the cellular membrane” and the possibility “that these conjugates may have more than one interaction site on the cellular membrane” [41].

#### 3.3.2. Phospholipid Membranes

The group of Paulo Almeida has, in several reports, characterized the interactions of transportan and analogs by stopped-flow fluorescence in phospholipid membranes. These studies, although they were carried out in phospholipid vesicles and using induced dye efflux, are of high impact in the characterization of CPP interactions and translocation mechanisms in live cells. Transportan 10 induced the graded release of the contents of phospholipid vesicles, as found by an analysis of their kinetics “by directly fitting to the data the numerical solution of mathematical kinetic models”. A global fit was obtained for a model in which “TP10 binds to the membrane surface and perturbs it because of the mass imbalance thus created across the bilayer”. This initiates the insertion of peptide monomers “transiently into its hydrophobic core and cross the membrane, until the peptide mass imbalance is dissipated” [163]. The studies of the kinetics and thermodynamics of TP10 (and its variants) binding and induced dye efflux in phospholipid vesicles showed that “the peptide-induced efflux becomes faster as the Gibbs energies for binding and insertion of the TP10 variants decrease” [134]. Later, the kinetics of the dye efflux induced by mastoparans, masL and masX from phospholipid vesicles showed the same graded kinetic model that we previously proposed for TP10 [164]. The mechanism of TP10W in model membranes (POPC) was shown to be “determined by the thermodynamics of insertion of the peptide into the lipid bilayer from the surface-associated state” [165]. It was shown that the translocation of the TP10W is “determined by the Gibbs energy of insertion into the bilayer from the membrane interface”, and that large effects on translocation are probably determined by hydrophobicity [166].

The quantitative detection of the entry of CF-TP10 into giant unilamellar vesicles (GUVs, DOPG and DOPC) containing Alexa Fluor 647 hydrazide showed first the increase of the fluorescence intensity of the GUV membrane, and at higher concentrations the leakage of AF647 was seen. It showed that “CF-TP10 can translocate across lipid bilayers without leakage of AF647, i.e., without pore formation”, “but prepores formed due to thermal fluctuation of the lipid bilayers are essential” [167].

### 3.4. Parallel Mechanisms of Endocytosis and Direct Translocation

In general, CPP cellular translocation is certainly initiated by the interaction with the components of the cell surface (e.g., phospholipids or cell surface proteins such as heparan sulfate) by electrostatic/hydrophobic interactions or hydrogen bonding, often between the guanidine of arginine side chains of cationic CPPs [2]. The initial CPP interactions will probably determine the cellular uptake route of the particular CPP or CPP/cargo conjugate. The possible uptake routes are, today, mainly divided into two general types: direct translocation and endocytosis, which likely depend on the homeostasis of the cells, and occur in parallel. The direct cellular translocation pathway was suggested in the first CPP studies [20] and was later confirmed by several studies. This energy-independent route has been explained by several experimental models, e.g., the inverted micelle model and the pore-formation carpet model etc. [2]. The involvement of several endocytic pathways was shown in CPP uptake mechanisms, especially in the case of CPP/cargo conjugates, e.g., macropinocytosis, and clathrin-mediated and caveolae/lipid-raft-mediated endocytosis [168]. It is likely that these conclusions hold even for the translocation mechanisms of transportan, as summarized below, both in artificial membranes and cells.

#### 3.4.1. Vesicles

The impact of membrane potential on the action of an AMP, lactoferricin B and TP10 in giant unilamellar vesicles (GUV) was suggested [169].

The primary amphipathicity of transportan and TP10 was shown to perturb and cause the leakage of the lipid membranes [170].

In GPMVs, the higher cholesterol content and tighter packing of the membranes reduces the accumulation of transportan, TP10 and MAP in the vesicles [171].

In model bilayers of POPC and POPG, R9, TP1, TP2 and TP3 showed that the hydrophilic and hydrophobic amino acids determine the interactions with phospholipids, and that the membrane rigidity defines the pore formation [172].

TP10 and melittin formed submicron pores in the lipid GUV membranes with the leakage of probes from the inside of the vesicles [173].

The interactions of Fl-R9, -Tat, -penetratin, -MAP, -transportan and -TP10 with giant plasma membrane vesicles (GPMVs) showed that amphipathic CPPs preferentially associate with liquid-disordered membrane areas, and that all of the tested CPPs accumulate into the lumen of GPMVs both at ambient and low temperatures, in conditions lacking endocytosis [138].

A molecular dynamics simulation study for the penetration of transportan across a DPPC bilayer yielded the free energy profile for the peptide inside the bilayer. It is energetically favorable for transportan to reside inside the bilayer because causing the higher ordering of the neighboring lipids initiates the reaching of “the other monolayer through the lysine residues”. After making the connection “between the two monolayers through the peptide, the bilayer thins significantly and the formation of a pore” is likely to happen [174].

In a giant unilamellar vesicle (GUV, DOPG, DOPC) TP10 induced the leakage of fluorescent probes, inducing pore formation in lipid membranes [175].

Translocating CF-TP10 into a giant unilamellar vesicle (GUV) entered the GUV lumen before pore formation in the membrane, showing the suppression of the translocation by cholesterol, translocating “across a bilayer through transient hydrophilic prepores in the membrane” [176].

CF-TP10 translocation into the lumen of single GUVs increased the membrane potential, φ_m_, up to 118 mV; it entered the GUV lumen without pore formation with an increased rate, with an increase in φ_m_ [177].

#### 3.4.2. Cells

In cells, both endocytosis and direct translocation have been demonstrated for the uptake of transportan and TP10.

Biotinyl–penetratin and –transportan showed, by indirect fluorescence with fluorescein-streptavidin detection in the Bowes melanoma cell line, that penetratin and transportan enter the cells by unrelated mechanisms, and that they do not belong to the same family of translocating peptides [139]. Transportan, TP10 and a translocation study across a Caco-2 human colon cancer cell layer showed a reversible decrease of the trans-epithelial electrical resistance of the barrier model, passing the epithelial cell layer mainly by a mechanism involving a transcellular pathway; penetratin did not affect the resistance of the cell layer to the same extent [118]. Confocal Raman microscopy (CRM) and atomic force microscopy (AFM) were used to study the infiltration and physiological effects of label-free transportan in SK-Mel-2 cells, showing the rapid entry (within 5 min) and widespread distribution of the peptide throughout the cytoplasm and the nucleus after ∼20 min, entering the cells via a nonendocytic mechanism with cytoskeletal changes triggered [140]. R9, Tat, transportan and TP10 showed interactions with glycosaminoglycans (GAGs) in the translocation of amphipathic CPPs [141]. The uptake of transportan and TP10 was not correlated to the presence of a receptor, energy or temperature, but when conjugated to cargoes, both by endocytosis and direct translocation [20,70], it was shown to be involved.

Several examples are available of delivery mechanism studies of cargos carried by transportan or analogs. The cellular delivery mechanisms of proteins (avidin or streptavidin) complexed with biotinylated transportan or TP10 have been studied, demonstrating cellular internalization by endocytosis. Fl-transportan and -TP10 complexed with Fl-avidin or -streptavidin-gold conjugates showed HeLa and Bowes cell transduction mostly by endocytosis with different pathways, but were also found in the perinuclear region as well as freely in the cytoplasm, suggesting endosomal escape [142]. Biotinyl–transportan, –oligoarginine and –Tat complexed to avidin–TexasRed in Cos-7 cellular uptake was induced by caveolin-dependent endocytosis [72]. Biotinyl–transportan and a biotinyl-TP10–avidin complex entered HeLa cells via the caveolin-1-dependent pathway [122]. Biotinyl–Tat, –transportan, and –pVEC complexed with avidin showed endocytotic, clathrin-dependent and independent endocytosis, a mechanism of peptide-mediated protein cellular transduction in HeLa cells with the partial depolarization of the plasma membrane [73].

Transportan-conjugated PNA oligomers also showed endocytotic cellular uptake in a few examples. A PNA_TAR_–transportan conjugate showed rapid cellular uptake by non-receptor-dependent endocytosis [143]. Tat-, penetratin- and transportan-S-S-PNA corrected the aberrant splicing in HeLa cells stably expressing luciferase with an aberrant splice site with a mainly endocytotic—in particular macropinocytotic—mechanism [46]. PNA conjugates of M918, penetratin, Tat, transportan, TP10, MAP and pVEC showed cellular translocation by an endocytotic route, as determined by well-known endocytosis inhibitors and tracers [47].

Transportan, complexed with a variety of NPs (AgNPs, AuNPs, IONPs and QDs) and BSA or dextran, labeled by fluorescent NHS-CF555 and NHS-CF647 dyes, showed in vitro and ex vivo (live tumor slices) cell entry via a receptor-dependent macropinocytosis process; “HSPGs and scavenger receptors are likely to be the cellular receptors” for this uptake [144].

#### 3.4.3. Mechanisms of PepFects and NickFects

PF3 and PF6 interaction studies with lipid membranes showed increased amphipathicity and the ability to insert into a lipid monolayer composed of zwitterionic phospholipids; the addition of “negatively charged phospholipids results in decreased binding and insertion of the stearylated peptides”. The trifluoromethylquinoline moieties in PF6 make no significant contribution to membrane binding and insertion. Interestingly, “TP10 actively introduces pores into the bilayers of large and giant unilamellar vesicles, while PF3 and PF6 do so only at higher concentrations”, suggesting their lower toxicity [145].

The membrane interactions of NF1 and NF51 with large unilamellar vesicles were studied by calcein leakage experiments showing membrane leakage by NF51, and not by NF1 and PF3.

The presence of pDNA cargo inhibited NickFect’s membrane perturbation; the “peptide alone causes membrane perturbation, but the cargo complex does not” [114].

The uptake by brain endothelial cells of the PF32/pDNA nanocomplexes is initiated via LRP-1 receptor-mediated endocytosis, as well as via scavenger receptor class A and B (SR-A3, SR-A5, and SR-BI)-mediated endocytosis [146].

The SR-A3 and SR-A5 were recruited after incubation with PepFect 14/SCO complexes, initiating the translocation of SR-As to the cell surface [147].

The coexistence of distinct molecular species (monomers, self-aggregates, peptide/oligonucleotide) in complexes of Fl-PF14/cyanine5-siRNA was studied by fluorescence correlation spectroscopy (FCS) and fluorescence cross-correlation spectroscopy (FCCS) in solution and at the plasma membrane of live cells. The ratio of the complex components varied with the pH, the peptide concentration and the proximity to the plasma membrane, suggesting “that the diverse cellular uptake mechanisms, often reported for amphipathic CPPs, might result from the synergistic effect of peptide monomers, self-aggregates and cargo complexes at the plasma membrane” [148].

The tracking of NF1 and NF51/pDNA complexes through endosomal transport was carried out by the separation of the endosomal vesicles by differential centrifugation. NF51 facilitates the rapid internalization of complexes into the cells; NF1 is less capable of inducing endosomal release, and a higher amount of complexes are routed to lysosomes for degradation [149].

#### 3.4.4. Toxicity

The knowledge of the toxicity window for a CPP or CPP/cargo in cells or tissues is a prerequisite for the in vitro and in vivo applicability of CPPs as drug delivery vectors. Due to the (often cationic) nature of CPPs, their toxic effect is caused by the specific interaction of CPPs with the membranes of cells and organelles, or with cell ingredients. The toxicity of CPPs has been observed only in a few reports [178] and it seems that, for transportan, at least some of its bioeffects can be explained by its in vitro and in vivo side effects, and not by its drug-delivery properties.

The comparison of the degradation half-lives of DOTA-conjugated CPPs in human serum showed that different CPPs degrade in very different time ranges; for example, for MAP, TP10, NLC the τ_1/2_ was >72 h; for penetratin it was 1.2 h; and for Tat it was 8.8 h [178]. This is important to consider if the degradation products of the CPPs carry side-effect sequences, and TP10 seems to be resistant.

Transportan and analogs on their own have been tested for toxicity in few cases, sometimes in comparison with additional CPPs. It was found [76] that TP10 caused cell death in HeLa and CHO cells at a dose of 20 μM, while Tat and penetratin did not influence the cell survival at concentrations. A series of TP10 analogues showed antimicrobial activities against multidrug-resistant bacteria, showing that the toxicity could be put to service as a therapeutic benefit. TP10 killed bacteria by membrane-active and DNA-binding activities, suggesting a use as a promising antibiotic candidate [179]. Penetratin, Tat, transportan and polyarginine showed that the toxicity correlated with the mitochondrial metabolic activity and cell viability [68]. MAP and TP10 caused significant cell membrane leakage in human cancer cell lines; penetratin, Tat and pVEC showed no significant toxicity or hemolytic effect on bovine erythrocytes [180]. Molecular dynamics suggested “that higher membrane disturbance leads to higher cellular uptake of peptides” in the case of TP10 [133]. TP10 actively introduced pores into the bilayers of large and giant unilamellar vesicles, while PF3 and PF6 did so only at higher concentrations, as is consistent with the lower toxicity of PF3 and PF6 observed in previous studies [145]. The toxicity of TP10 and MAP in *Neisseria meningitides* [181] and transportan in *Mycobacterium tuberculosis* [182] has been reported.

The delivery of cargo by transportan is very efficient in many cells and also in vivo. The side effects of such delivery have been addressed in several reports. Human IFN-gamma fused with penetratin showed the decreased toxicity of IFN-gamma due to the efficient delivery [183]. Tat, penetratin and transportan, and their conjugates with BSA cargo showed no toxicity or initiation of an immune response in epithelial cells [78]. TP6, TP7 and R9 showed significant cellular toxicity above 3−5 μM in TP–PNA conjugates [49]. Stearoyl–TP10 showed the efficient delivery of a splice-correcting 2′-OMe RNA ONs for functional splice correction with no toxicity; stearoyl–R9 and –penetratin did not improve the transfection [92]. Stearoyl-TP10/plasmid nanoparticles entered different cell types with high gene expression levels with no toxicity and no nonimmunogenecity in vivo when administred intramuscularly or intradermally [60]. Analogs of TP10, TK and TH with Lys residue replaced by His showed membrane translocation with lower toxicity than TP10; the TH–camptothecin conjugate “exhibited remarkable cytotoxicity to cancer cells in a pH-dependent manner” [150]. TP10 and PepFects (PF3, PF4, and PF6) and their complexes with plasmid and siRNA showed no effect on the cytotoxic and immunogenic response, e.g., IL-1β, IL-18 and TNF-α cytokine release, cell viability, and apoptosis in vitro and in vivo [151]. TP, TP10, TP–biot1, TP–biot13 and TP10–biot1 conjugates with FITC–streptavidin and siRNA in the HT29 and HCT116 cell lines showed no significant cytotoxic effect at 0.5–5 µM [75]. Transportan analog T9(dR) complexed with siRNA (against a nucleoprotein (NP) gene segment of the influenza virus) showed the delivery of siRNA into 293T, MDCK, RAW and A549 cells with low cellular toxicity, and in mice infected with the PR8 influenza virus when they were given a combined tail vein injection of siNP and T9(dR) or TP; all of the mice survived and showed weight recovery at 2 weeks post-infection [57]. Chloroquine–TP10 conjugates showed higher antiplasmodial activity than the parent TP10 “at the cost of an increased hemolytic activity” seeming “unsuitable for safe intracellular delivery of antimalarial aminoquinolines due to hemolysis issues” [152]. Conjugates of TP10 with ciprofloxacin or levofloxacin (fluoroquinolone antibacterial agents) showed antifungal in vitro activity against human pathogenic yeasts of the *Candida* genus, showing cytotoxicity against the HEK293, HepG2 and LLC-PK1 cells causing the intrinsic cytoplasmic membrane disruption activity [153].

### 3.5. Metabolomics and Transcriptomics

Genomic, transcriptomic and proteomic data is increasing in life sciences, showing growing potential for integrative proteogenomic data analyses in these areas. This analysis enables us to discover novel pathways of biochemical processes as well as the involvement of novel proteins and genes, improving our understanding of biological processes [184]. These tools are applied in a few cases in order to understand the novel CPP mechanisms, including transportan, and its modifications and analogs.

Metabolomics studies yield “the unique chemical fingerprints that specific cellular processes leave behind”, identifying the small-molecule metabolite profiles [185], and could be called a functional readout of the genome, a functional genome, or a proteome [186]. We compared the alterations in the cytosolic metabolome of CHO cells caused by exposure to transportan, penetratin, Tat, R9 and MAP, showing that the “intracellular metabolome was the most affected by transportan followed by Tat and MAP”; transportan mostly affected the cellular redox potential, and depleted energy and the pools of purines and pyrimidines [187]. It remains to connect these data to the functionality of transportan in cargo delivery.

Professor Jim Eberwine’s group introduced the PAIR (PNA-assisted identification of RBPs and RNA binding proteins) method for the identification and dynamics of the RNA-RBP interactions in live cells, patent number 8632972, which was further developed by the University of Pennsylvania. It applies transportan–PNA (12–18 ONs) with a photo-activatible label, targeting in live cells mRNA and, after UV light stimulation, crosslinks the PNA–Bpa–RBP complexes, which after purification can be identified by MS “in order to evaluate essential regulatory proteins that control all modes of RNA processing and regulation” [42,188,189,190,191].

Real-time transcriptome in vivo analysis (TIVA) was obtained by the application of transportan–TIVA–tag [192,193], delivered to the cytoplasm in vivo, enabling us to target and isolate cell-specific transcriptomes upon photoactivation. The method permitted us to yield the transcriptomic landscape of individual cells in mouse brains.

Following time course treatments with penetratin, PepFect14, mtCPP1 and TP10, HeLa cells were transcriptionally profiled by RNA sequencing, showing the response of “specific sets of genes related to ribosome biogenesis, microtubule dynamics and long-noncoding RNAs being differentially expressed compared to untreated controls” [194].

PF6/siRNA nanoparticles showed robust RNAi responses in various cell types with minimal associated transcriptomic or proteomic changes, and promoted strong RNAi responses in different organs following systemic delivery in mice without any associated toxicity [27]. This work is a good example where the transcriptomics analysis enables us to decide about the side effects of ON transfection.

## 4. Targeting

**Figure 1:**
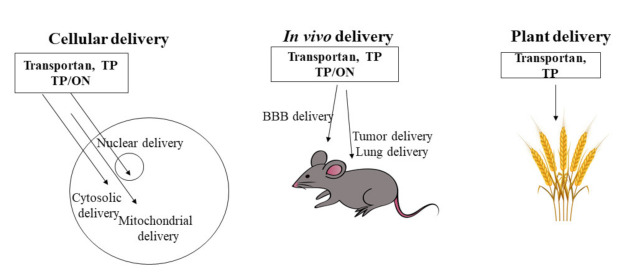
Schematic summary of the reported applications of transportan and its analogs with specific targeting.

The efficient cellular uptake of CPPs in most cell lines in vitro has been demonstrated repeatedly, although some examples exist in which a certain CPP is more efficient in certain (cancer) cells, and where the PepFects vectors are able to transfect “difficult” cell lines exclusively [2,27]. In vivo, the lack of cell/organ specificity has been demonstrated, following any type of administration. In drug development, this suggests non-desired off-target side-effects and, hence, the intensive development of the targeted delivery of CPPs and their conjugated cargos is ongoing. The field is fueled by the broad interest in cancer targeting in vivo, e.g., by the application of the tumor-targeting antibodies, the targeting of specific cell surface receptors or antigens at tumor sites, the targeting of subcellular organelles with, e.g., NLS mitochondrial targeting sequences using the activatable CPP (aCPP) strategy. Below, I summarize the few reports which are available for the targeting of transportan and its modified versions using these strategies; see Figure 1.

Transportan, like some CPPs, showed nuclear targeting by itself [20]; see Figure 1. Later, the transportan analogues PepFects and NickFects showed powerful DNA nuclear delivery properties. The nuclear uptake of a double-stranded oligonucleotide NF-kappaB decoy ON in rat primary glial cells was facilitated by noncovalent binding to transportan 10 via a complementary PNA sequence [195,196].

Transportan showed a high translocation efficiency in plants, e.g., tobacco protoplasts [119]. The permeabilization of the cell wall increased the uptake of Fl–Tat, –Tat_2_, -mutated–Tat, –pVEC and –transportan in immature wheat embryos [59]. Several CPPs were able to translocate into algal cells: FITC–pVEC, –Tat, –penetratin and –transportan translocated into *Chlamydomonas reinhardtii* [197], and the pVEC, Tat, penetratin and transportan translocated into algal cells [197]

Few attempts have been made to study and develop BBB-passing delivery; see Figure 1. Transportan could effectively cross the Caco-2 cell layer with a trans-cellular mechanism in a two-chambered Transwell model system [118], suggesting the possibility for a BBB-passing delivery system. The in vivo BBB transport characteristics of pVEC, SynB3, Tat, TP10 and TP10-2 showed a low brain influx for transportan, but the significant labeling of the brain parenchyma by the others, which was not correlated with their CPP properties [124]. pVEC, SynB3 and Tat 47–57 showed very high unidirectional BBB influx rates, whereas TP10 and TP10-2 showed a negligible-to-low brain influx with no significant efflux out of the brain, except for pVEC [124]. Myristoyl–transportan–Tf with encapsulated siRNA showed targeted delivery through BBB with functional gene silencing in vitro [99]. A TP10–vancomycin conjugate showed antibacterial activity and crossed the BBB in a mouse brain after i.v. administration [198]. A TP10–dopamine conjugate showed the penetration of the BBB and access to the brain tissue with anti-parkinsonian activity (higher than that of l-DOPA), “exhibiting high affinity to both dopamine D_1_ and D_2_ receptors” (in the case of D_1_, a much higher activity than that of DA) [124].

The PepFect delivery strategy of ONs for drug delivery across the BBB has been attempted, although these studies are still at the in vitro level; see Figure 1. PF32 with the targeting ligand angiopep-2 (binding to the endothelial LDLR-related protein-1, LRP-1), showed complexed pDNA delivery in an in vitro Transwell model of the BBB through receptor-mediated endocytosis via scavenger receptors class A and B (SCARA3, SCARA5 and SR-BI) [146]. PF14 and PF28, modified covalently with BBB targeting peptides, and angiopep-2 modified by covalent conjugation and complexed with siRNA_Luc_ showed specific gene-silencing efficiency in human glioblastoma cells U87 MG-luc2, as compared to non-glioma targeted cells [199].

Cancer-targeting is often the subject of CPP delivery development; see Figure 1. iRGD- and CPP (myristoyl-transportan)-grafted PEG backbone structure NPs were shown to carry siRNA into mammalian cells, silencing the RNA target in a mouse model of cancer [200]. TP10–SRC1LXXLL with the peptide from human SRC-1 nuclear receptor box 1, containing an LXXLL motif, induced the dose-dependent cell death of breast cancer cells by reducing the viability and proliferation of breast cancer MDA-MB-231 cells and dermal fibroblasts, suggesting it as a drug candidate in the treatment of cancers [201].

Due to its high efficacy, PepFect- and NickFect-assisted ON delivery has earned attention in the development of future cancer gene therapies by specific targeting. It is highly valuable that these transfection-targeting experiments are available on animal models in vivo.

Using the i.v. administration route in vivo, PEG- and MMP substrate-functionalized PF14 (aCPP approach) complexed with pDNA showed the efficient induction of gene expression specifically in tumors, avoiding normal tissues [97].

NF55/pDNA nanoparticles showed the transfection of the majority of cells and in vivo specific tumor transfection in various mouse tumor models, including an intracranial glioblastoma model [30].

MMP-2/-9 activatable PF144/pDNA nanocomplexes for anti-angiogenic gene (encoding short hairpin RNA) delivery showed the inhibition of tumor growth by silencing the vascular endothelial growth factor (VEGF) expression in orthotopic 4T1 breast tumor-bearing mice [202].

PF14 and NF55 preferentially transfect lung tissue upon their systemic administration with the complexed siRNA and pDNA encoding shRNA against cytokine TNFα in models of acute lung inflammation and asthma in mice, and showed efficient anti-inflammatory effects in both disease models [203].

PF14, covalently conjugated to mitochondrial-penetrating peptide, mtCPP1, complexed with ONs affected biological functions both in the cytoplasm and on the mitochondria, suggesting “the potential to be used as a treatment for patients with mitochondrial disorders” [204].

## 5. Conclusions

Transportan and its modified versions—e.g., TP10, PepFects and NickFects—are widely used as efficient delivery vectors of a wide range of cargos, such as small molecules, peptides and proteins, as well as oligonucleotides such as short ONs, siRNA, miRNA, decoy ON, plasmids and mRNA. These various examples of applications have been used in studies of CPP mechanisms as well as for the development of therapies and the diagnosis of diseases.

Remarkably, PepFects and NickFects have demonstrated the ability to form stable nanoparticles with the very efficient transfection of ONs in vitro and in vivo, paving a way to future gene therapy. The addition of these CPPs to the available nanoparticle platforms may, in the future, contribute to novel, improved drug delivery systems.

Transportan and its versions have been modified in order to achieve the controlled targeted delivery of bioactive cargos, especially for future cancer gene therapy. For that, the detailed knowledge of CPP mechanisms, toxicity, immunogenicity, efficiency and kinetics should be achieved, and this work is ongoing.

## Figures and Tables

**Table 1:** Summary of the mechanisms of uptake and toxicity studies of transportans and their conjugates with bioactive cargos; see the details in the text.

TP Analog Used	System	Result in Brief
Visualization		
N-ε13-biotinyl-TP/Fl-streptavidin, ^125^I-TP	fluorescence microscopy, gamma-counting	visualization in the fixated Bowes cells [20,36]; cellular internalization of TP is not an artefact caused by cell fixation
biotinyl-, Fl-, Abz-TP, -TP10	fluorescence microscopy, Transwell	visualization of crossing a Caco-2 human colon cancer cell layer in vitro by a transcellular pathway [118]
Fl-transportan antisense conjugates	confocal microscopy	mainly endocytic, macropinocytotic pathway for cellular uptake [46]
TP	FACS, spectrofluorimetry	protein uptake by endocytotic mechanism in HeLa cells of CPP-avidin complexes [73]
Fl-TP, -TP10	fluorescence microscopy, spectrofluorometry and FACS	visualization in different mammalian and plant cells [119]
TP	FACS	similar kinetic uptake profiles in HeLa, A549 and CHO cell lines [68]
^111^In- or ^68^Ga-TP	micro-PET imaging	uptake by six tumor cell lines and biodistribution in PC-3 tumor-bearing nude mice [120]
TP	fluorescence polarization, quenching and CD spectroscopy	in small phospholipid vesicles the helical penetratin and transportan lie along the vesicle surface, penetratin variants appear to penetrate deeper into the membrane [121]
AU NP-functionalized TP- and TP10-protein	TEM	cell entry mechanisms and intracellular trafficking of constructs were studied [122]
myristoylated TP-Tf	fluorescence images and functional gene silencing by siRNA	targeting of siRNA over BBB [99]
TAMRA-TP10	fluorescence microscopy, cell toxicity	complexes of with cisplatin (cPt) in (HEK293, HEL299, HeLa OS143B cell lines were visualized [123]
Alexa488-TP10	flow cytometry, live-cell imaging and image analysis	showed that the glycine-phenylalanine switch was most dramatic in TP10 [79]
^125^I-TP10 and -TP10-2	in vivo injection	BBB delivery [124]
TP10	spheroid model of the BBB, ex vivo imaging	showed increased delivery to mouse brains [125]
TP- isoniazid	Langmuir balance technique and AFM imaging	conjugates showed similar internalization rate into EBC-1 human squamous cell carcinoma in imaging of penetrated lipid layers [126]
**Structure and interactions of transportan**		
TP	CD	random coil structure in water, in SDS micelles 60% induced helix [127]
TP	CD	60% alpha-helix in phospholipid vesicles [128]
TP	CD	helical structure in small phospholipid vesicles [121]
TP	NMR	in neutral bicelles alpha-helix in the C-terminus and tendency to form an alpha-helix in the N-terminus [129]
TP	CD	structure in neutral DMPC bicelles and negative DMPG-containing bicelles different from each other [130]
TP	CD	obtain amphiphilic α-helix when bound to membranes of vesicles composed of typical eukaryotic lipids [131]
TP10	solid-state ^19^F-NMR, CD	a range of conformations in the DMPC/DMPG vesicle membrane-bound state, C-terminal α-helix is embedded in the membrane being tilted [132]
TP10 and 5 analogs	molecular dynamics simulation	forming α-helical conformation, the higher membrane disturbance yields higher cellular uptake in cells [133]
TP	CD	increased membrane affinity with DPPC and DPPC + mycolic acid mixed monolayers [126]
TP10	Gibbs energy studies	peptide-induced efflux, becoming faster with decrease of the Gibbs energies for binding and insertion [134]
TP and analogs	PepLook algorithm	peptide polymorphism showed common conformational polymorphic characteristics [135]
Cys-TP	EPR	in DMPC/cholesterol caused lipid ordering and a large increase in permeation [136]
TP10	molecular dynamics	interactions with POPC bilayer initiated α-helix with hydrophobic side facing the hydrophobic lipid core [137]
TP10	confocal microscopy	interaction of GPMVs revealed association with liquid-disordered membrane areas [138]
PF6	DLS	mean diameter of PF6/siRNA NPs was shown to be below 200 nm [27]
PF14	DLS	PF14/SCO NPs possessed a net negative charge [28]
PFs	CD, DLS, QSAR	PFs/Luc-plasmid NPs for study of structural requirements for cell penetration [95]
His-PFs, PF132	DLS, CD, calcein leakage	complexes formed were small at pH 7 and grew under acidic conditions [96]
**Parallel mechanisms of endocytosis and direct translocation in cells**		
biotinyl-TP	fluorescence microscopy	cellular uptake by unrelated mechanisms [139]
TP, TP10	Transwell model	translocation across a Caco-2 human colon cancer cell layer by transcellular pathway [118]
TP	CRM and AFM	entered SK-Mel-2 cells within 5 min and widespread distribution via a nonendocytic mechanism [140]
TP, TP10	confocal laser scanning microscopy, GMPV	showed the interactions with glycosaminoglycans [141]
TP, TP10	fluorescence microscopy	uptake, when conjugated to cargoes, involved both endocytosis and direct translocation [20,70]
Biotinyl-TP, -TP10/avidin	fluorescence microscopy	cellular internalization in HeLa and Bowes cellc by endocytosis with different pathways [142]
biotinyl-TP, -TP10/avidin	confocal microscopy	entered Cos-7 cells by caveolin-dependent endocytosis [72]
biotinyl-TP, -TP10/avidin	confocal microscopy	entered HeLa cells via caveolin-1-dependent pathway [122]
biotinyl-TP, -TP10/avidin	FACS analysis and spectrofluorimetry	clathrin-dependent and -independent endocytosis uptake in HeLa cells with partial depolarization [73]
TP-PNA	fluorescence microscopy	rapid cellular uptake by non-receptor-dependent endocytosis [143]
TP-PNA	splice correction assay	splice correction in HeLa/Luc cells with mainly endocytotic, particular macropinocytotic mechanism [46]
TP-, TP10-PNA	splice correction assay	showed cellular translocation by endocytosis [47]
TP/NPs, /BSA, /dextran	fluorescence microscopy	complexed with NPs showed in vitro and ex vivo cell entry via a receptor-dependent macropinocytosis process [144]
**Mechanisms of PepFects and NickFects**		
PF3, PF6	interaction studies with lipid membranes	increased amphipathicity and their ability to insert into a lipid monolayer composed of zwitterionic phospholipids [145]
NFs/pDNA	membrane perturbation study	pDNA cargo inhibited membrane perturbation by NFs [114]
PF32/pDNA	Transwell model	uptake by brain endothelial cells via LRP-1 receptor-mediated endocytosis and scavenger receptors [146]
PF14/SCO	fluorescence microscopy	SR-A3 and SR-A5 recruited by PF14/SCO complexes [147]
PF14/Cy5-siRNA	FCS, FCCS	coexistence of monomers, self-aggregates of peptide/ON in complexes in solution and at the plasma membrane of live cells [148]
NF1/, NF51/pDNA	analysis of separated endosomal vesicles by differential centrifugation	NF51 facilitates rapid internalization of complexes into the cells, NF1 is less capable to induce endosomal release [149]
**Delivery of the cargo**		
TP-BSA	toxicity study	showed no toxicity or initiation of an immune response in epithelial cells [78]
TP6, TP7-PNA	toxicity study	significant cellular toxicity above 3−5 μM in TP-PNA conjugates [49]
St-TP10/SCO	splice-correcting assay, toxicity study	delivery of SCOs for functional splice correction with no toxicity [92]
St-TP10/plasmid	toxicity study, gene expression in vivo	entered different cells with high gene expression level with no toxicity and no nonimmunogenecity in vivo [60]
TK- and TH- camptothecin	toxicity study	TH-camptothecin showed cytotoxicity to cancer cells [150]
TP10, PFs/plasmid, /siRNA	toxicity study	peptide/plasmid and /siRNA showed no cytotoxic and immunogenic response, in vitro and in vivo [151]
TP, TP10, TP-biot1, TP-biot13, TP10-biot1	toxicity study	no significant cytotoxic effect at 0.5–5 µM [75]
T9(dR)/siRNA	cells, in vivo toxicity	showed cellular delivery of siRNA and in mice infected with PR8 influenza virus, and antiviral activity [57]
chloroquine-TP10	antimalarial activity	higher antiplasmodial activity in safe delivery of antimalarial aminoquinolines [152]
TP10-ciprofloxacin or -levofloxacin	antifungal in vitro activity	TP10-ciprofloxacin or -levofloxacin showed antifungal in vitro activity against human pathogenic yeasts [153]

## Data Availability

The study did not report any original data.

## Abbreviations

aa	amino acid(s)
Abz	aminobenzoic acid
AFM	atomic force microscopy
AMP	antimicrobial peptide
AS	antisense
ASO	antisense oligonucleotide
BBB	blood-brain-barrier
BSA	bovine serum albumin
CD	circular dichroism
CF-	carboxyfluoresceinyl
Chol	cholesteryl
CPP	Cell-penetrating peptides, e.g., pVEC, SynB3, CADY etc.
CRM	confocal Raman microscopy
DLS	dynamic light scattering
DMPC	1,2-dimyristoyl-sn-glycero-3-phosphocholine
DMPG	1,2-dimyristoyl-sn-glycero-3-phosphoglycerol
DPPC	dipalmitoylphosphatidylcholine
FACS	Fluorescence-activated cell sorting
Fl-	fluorescently labelled
GFP	green fluorescent protein
GPMV	giant plasma membrane vesicles
GUV	giant unilamellar phospholipid vesicles
LNA	locked nucleic acid
Luc	luciferase
LUVs	large unilamellar phospholipid vesicles
MAP	membrane active peptide, CPP
miRNA	microRNA
NF	NickFect, e.g., NF51 and NF55
NLS	nuclear localisation signal
NMR	nuclear magnetic resonance
NP	nanoparticle
ON	oligonucleotide
O	ornithine
pDNA	plasmid
PEI	polyethyleneimine
PET	Positron emission tomography
PF	PepFects, e.g., PF6, PF14
PMO	phosphorodiamidate morpholino ON
PNA	peptide nucleic acids
POPC	1-palmitoyl-2-oleoyl-sn-glycero-3-phosphocholine
PS	phosphothioate ON
QSAR	quantitative structure-activity relationhip
R9	Arg_9_
SCO	splice correctin ON
shRNA	short hairpin RNA
siRNA	short interfering RNA
TAMRA	5-(and-6)-carboxytetramethylrhodamine
TEM	transmission electron microscopy
TP	transportan
TP10	transportan 10
Tf	transferrin
